# Cardiometabolic Risk Profiles Associated with Chronic Complications in Overweight and Obese Type 2 Diabetes Patients in South China

**DOI:** 10.1371/journal.pone.0101289

**Published:** 2014-07-03

**Authors:** Yanzhen Cheng, Hua Zhang, Rongping Chen, Fan Yang, Wei Li, Lishu Chen, Shaoda Lin, Ganxiong Liang, Dehong Cai, Hong Chen

**Affiliations:** 1 Department of Endocrinology, Zhujiang Hospital of Southern Medical University, Guangzhou, China; 2 Department of Endocrinology, Affiliated Hospital of Gulin Medicine University, Guilin, China; 3 Department of Endocrinology, Wu Jing Zong Dui Hospital of Guangdong Province, Guangzhou, China; 4 Department of Endocrinology, The Second Affiliated Hospital of Shantou University Medicine College, Shantou, China; 5 Department of Endocrinology, The first Affiliated Hospital of Shantou University Medicine College, Shantou, China; 6 Department of Endocrinology, People's Hospital of Zhongshan City, Zhongshan, China; University of Barcelona, Faculty of Biology, Spain

## Abstract

**Background:**

Type 2 diabetes is often accompanied by altered cardiometabolic risk profiles, including abdominal obesity, hypertension, and dyslipidaemia. The association of altered cardiometabolic risk profiles with chronic complications of diabetes is not well investigated.

**Methods:**

We recruited 2954 type 2 diabetes patients with a body mass index ≥25 kg/m^2^ who visited the diabetes clinics of 62 hospitals in 21 cities in Guangdong province of China from August 2011 to March 2012. Demographic characteristics, personal and family medical histories, and data on chronic complications of diabetes were collected. Clinical examinations and laboratory assessment were conducted.

**Results:**

Abdominal obesity was found in 91.6% of the study population, elevated blood pressure in 78.3%; elevated serum triacylglycerols in 57.8%, and reduced serum HDL-C in 55.9%. Among the cardiometabolic risk factors, elevated blood pressure was significantly associated with almost all the chronic complications of diabetes. After adjusting for age, gender, duration of diabetes, and HbA1c, elevated blood pressure was significantly associated with diabetic retinopathy (OR 1.63, 95% CI: 1.22–2.19), diabetic nephropathy (OR 3.16, 95% CI: 2.25–4.46), cardiovascular disease (OR 2.71, 95% CI: 1.70–4.32), and stroke (OR 1.90, 95% CI: 1.15–3.12). Abdominal adiposity was significantly associated with diabetic nephropathy (OR 1.39, 95% CI: 1.11–1.74). Elevated triacylglycerols was significantly associated with diabetic retinopathy (OR 1.29, 95% CI: 1.05–1.58) and diabetic nephropathy (OR 1.30, 95% CI: 1.05–1.58). Reduced HDL-C was significantly associated with stroke (OR 1.41, 95% CI: 1.05–1.88).

**Conclusions:**

Altered cardiometabolic risk profiles, and elevated blood pressure in particular, were significantly associated with chronic complications in overweight and obese patients with type 2 diabetes. Future studies on the prevention of chronic complications of diabetes might make lowering blood pressure a primary target.

## Introduction

Rapid economic growth, acceleration of urbanization, and lifestyle changes are resulting in an increase in the prevalence of type 2 diabetes. In China, the most recent estimate of the overall prevalence of diabetes in the adult population is 11.6%, the majority being type 2 diabetes [1]. Type 2 diabetes and its related complications have a heavy economic burden on families and the health system, which is increased by the development of altered cardiometabolic risk profiles, including abdominal obesity, increased blood pressure and dyslipidaemia. Clustering of these cardiometabolic risk factors, known as metabolic syndrome (MetS) [2], [3], has been reported in various populations, and its prevalence has increased over the past decades. For example, the National Health and Examination Survey (NHANES) has shown that the prevalence of MetS in the United States increased over time from approximately 29.2% in 1988–1994 to 34.6% in 1999–2002 [4]. A cross-sectional survey conducted in 2000–2001 in a nationally representative sample of 15 540 Chinese adults 35–74 years of age found that the age-standardized prevalence of MetS was 9.8% in men and 17.8% in women [5].

Limited but increasing data suggest a link between altered cardiometabolic risk profiles and diabetic chronic complications [6]. A recent multinational study in patients with type 2 diabetes showed high levels of low-density lipoprotein cholesterol (LDL-C), triacylglycerols, and increased systolic blood pressure were positively associated with both macrovascular and microvascular complications of diabetes. Conversely, a high level of high-density lipoprotein cholesterol (HDL-C) was negatively associated with macrovascular complications [7]. In the Verona Diabetes Complications Study [8], MetS in type 2 diabetes was associated with a fivefold increase in cardiovascular disease (CVD) risk, and obese subjects with MetS had an adjusted relative risk (RR) for CVD of 2.13 after 11 years of follow-up [9]. Type 2 diabetes patients who are overweight or obese (body mass index ≥25 kg/m^2^) are at increased risk of chronic complications of diabetes, which is consistent with the observation that several metabolic characteristics including inflammation and insulin resistance are common to MetS, obesity and type 2 diabetes [10],[11]. Unfortunately previous epidemiological investigations lack data on the association of altered cardiometabolic risk profiles with chronic complications of type 2 diabetes in patients who are overweight or obese. The objective of this collaborative, multicentre study in South China was to investigate those associations.

## Materials and Methods

### Subjects

This study enrolled overweight and obese patients who visited the diabetes clinics at 62 hospitals in 21 cities in Guangdong province of China from August 2011 to March 2012. The study was approved by the Human Research Ethics Committees of Zhujiang Hospital and performed in accord with the ethical principles of the Declaration of Helsinki [12]. Written informed consent was obtained from each patient. Eligible patients were identified using the World Health Organization (WHO) diagnostic criteria for diabetes [13] or by the current use of antidiabetic agents, were diagnosed with type 2 diabetes at least 6 months previously, and had a body mass index (BMI) ≥25 kg/m^2^. Overweight or obesity was defined as a BMI ≥25 kg/m^2^, and calculated as body weight divided by body height squared [14]. Patients whose age at diagnosis was <18 years and those with type 1 diabetes, retinal, kidney, neurological, or cardiovascular disease (CVD), were excluded.

### Data collection

Demographic characteristics, personal and family medical histories, and information on diabetic complications were collected using a standard questionnaire administered by trained staff. Weight was measured with a portable scale while the individual was lightly dressed and without shoes, Height was measured with a portable, rigid measuring rod, and mean blood pressure (BP) was calculated from two measurements taken in a sitting position after 10 min of rest. Blood samples were collected for analysis of plasma glucose, haemoglobin (Hb)A1c, total cholesterol, HDL-C, LDL-C, and triacylglycerols.

The cut-off points of altered cardiometabolic risk factors were defined according to the modified National Cholesterol Education Programme (NCEP) Expert Panel on Detection, Evaluation, and Treatment of High Blood Cholesterol in Adults criteria (Adult Treatment Panel III) [2], [3]. Abdominal obesity was defined as waist circumference >90 cm (men) or >80 cm (women); elevated blood pressure was defined as systolic/diastolic blood pressure ≥130/85 mmHg; elevated triacylglycerols was defined as serum triacylglycerol concentration ≥1.70 mM; and reduced HDL-C was defined as HDL-C concentration <1.40 mM (men) or <1.30 mM (women). Diabetic retinopathy (DR) was assessed by fundus photography and graded as absent, non-proliferative, or proliferative. The presence of microalbuminuria, a urinary albumin excretion rate (UAER) of 20–200 µg/min or macroalbuminuria (UAER >200 µg/min) was included in the analysis of diabetic nephropathy (DN). Diabetic peripheral neuropathy (DPN) was diagnosed as abnormal sensory perception of a 10 g Semmes–Weinstein monofilament at the hallux of each foot. CVD was defined as experiencing typical chest pain, having a history of previous myocardial infarction, or presence of pathological changes on electrocardiogram testing. Cardiac arrhythmia, peripheral vascular disease, and atherosclerosis were not included as CVD. Ischaemic or haemorrhagic strokes were identified in the previous medical history or from pathological changes on imaging examination.

### Statistical analysis

All statistical analyses were done using SPSS version 13.0 (SPSS Inc., Chicago, IL, USA). Data were presented as means±standard deviation (SD) or percentages, as appropriate. Descriptive statistical analyses were carried out with either Student's *t*-test or one-way analysis of variance (ANOVA), and the chi-square test was used to compare categorical variables. Multivariate logistic regression analysis was performed to identify risk factors. A two-tailed *p*<0.05 was considered statistically significant.

## Results

A total of 2954 patients were included in this study, and the clinical characteristics of the participants are shown in Table 1. The average age of the study population was 58.5±13.1 years, and was higher in women (61.2±11.6 years) than in men (55.7±13.9 years). Women also had a longer duration of diabetes, higher BMI and systolic blood pressure, smaller waist circumference and lower diastolic blood pressure. Women had higher total cholesterol, HDL-C, and LCL-C levels, but lower triacylglycerol levels. There was no significant gender difference in HbA1c level.

**Table 1:** Characteristics of participants.

Variables	Overall	Men	Women	*P* *
Age (years)	58.5±13.1	55.7±13.9	61.2±11.6	<0.001
Duration of diabetes (years)	7.0±6.3	6.1±5.8	7.8±6.7	<0.001
BMI (kg/m^2^)	27.9±2.7	27.8±2.5	28.1±2.8	0.001
Waist circumference (cm)	96.7±9.1	98.0±8.9	95.3±9.1	<0.001
HbA1c(%)	8.7±2.3	8.7±2.4	8.6±2.2	0.195
SBP (mmHg)	136.5±18.9	134.6±18.2	138.3±19.3	<0.001
DBP (mmHg)	80.5±10.8	81.0±10.7	79.3±10.8	<0.001
TC (mM)	5.28±1.53	5.15±1.61	5.40±1.44	<0.001
Triacylglycerols (mM)	2.40±1.81	2.54±1.98	2.27±1.50	<0.001
HDL-C (mM)	1.20±0.63	1.14±0.73	1.26±0.50	<0.001
LDL-C (mM)	3.06±1.16	2.98±1.19	3.14±1.10	<0.001

*one-way ANOVA.

BMI, body mass index; HbA1c, haemoglobin A1c; SBP, systolic blood pressure; DBP, diastolic blood pressure; TC, total cholesterol; LDL-C, low-density lipoprotein cholesterol; HDL-C, high-density lipoprotein cholesterol.

**Figure 1:**
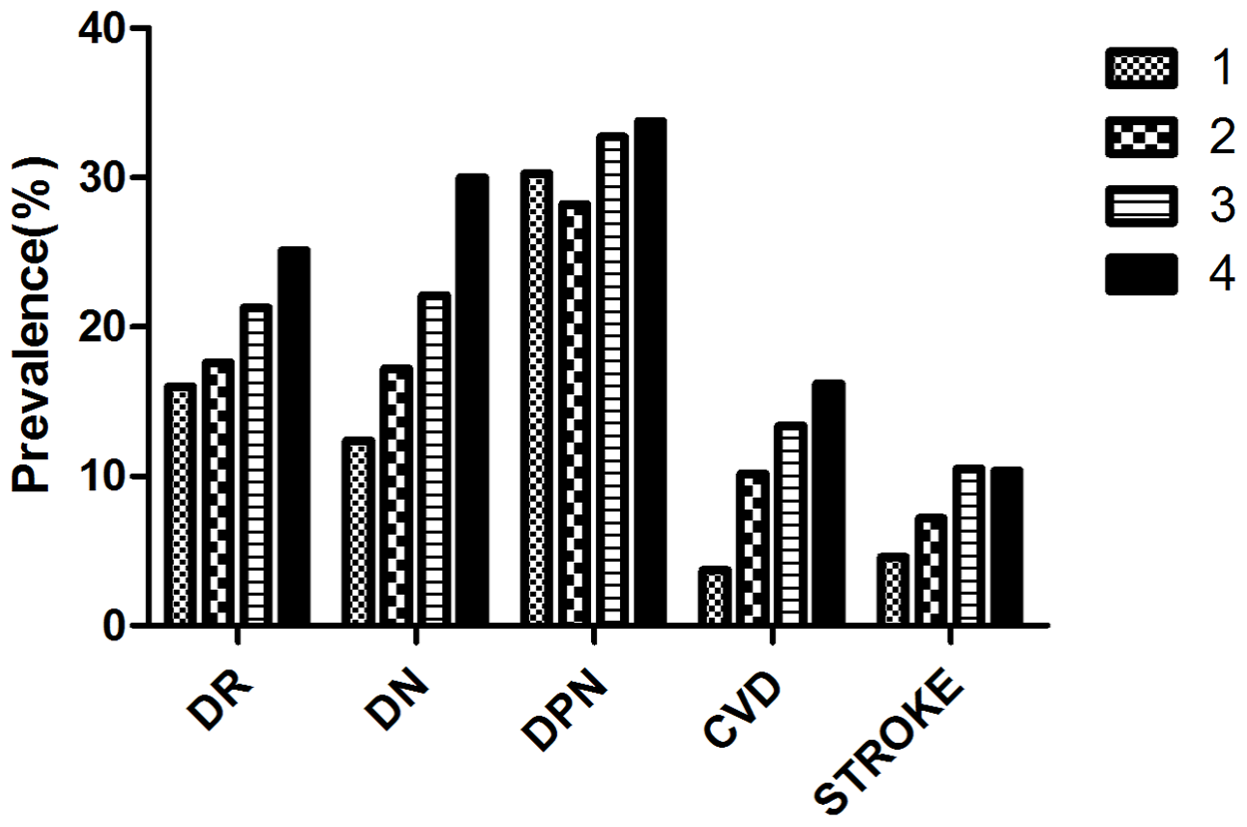
Prevalence of diabetic chronic complications according to the number of altered cardiometabolic risk factors. 1, type 2 diabetes plus any other one component; 2, type 2 diabetes plus any other two components; 3, type 2 diabetes plus any other three components; 4, type 2 diabetes plus any other four components; DR, diabetic retinopathy; DN, diabetic nephropathy; DPN, diabetic peripheral neuropathy; CVD, cardiovascular disease.

As shown in Table 2, 91.6% of participants were found to be obese, 78.3% had elevated BP, 57.8% had elevated serum triacylglycerols and 55.9% had reduced serum HDL-C. The prevalence of increased waist circumference, elevated BP and reduced serum HDL-C was higher in women than in men (*P<*0.001). The prevalence of chronic complications ranged from 9.2% for stroke to 31.6% for DPN (Table 3). Fig.1 shows the influence of cardiometabolic risk factors on the prevalence of chronic complications, indicating that increase in the number of risk factors was associated with increase in the prevalence of chronic complications. For example, the prevalence of DR associated with one, two, three, and four cardiometabolic risk factors was 16.0%, 17.6%, 21.3%, and 25.1%, respectively (*P = *0.001). The corresponding values for DN were 12.4%, 17.2%, 22.1%, and 30.0%, (*P*<0.001); for CVD, 3.7%, 10.2%, 13.4%, and 16.2%, (*P*<0.001); and for stroke, 4.6%, 7.2%, 10.5%, and 10.4% (*P* = 0.006). There were no statistically significant differences in DPN prevalence with increase in the number of cardiometabolic risk factors (30.3%, 28.2%, 32.7%, and 33.8%, respectively; *P* = 0.091).

**Table 2:** Prevalence of altered cardiometabolic risk factors.

Cardiometabolic risk factors	Men		Women		*P* *
	*n*	%	*n*	%	
Individual cardiometabolic risk factor					
Abdominal obesity	1233	84.1	1474	99.1	<0.001
Elevated BP	1107	75.5	1205	81.0	<0.001
Elevated triacylglycerols	856	50.1	851	57.2	0.527
Reduced HDL-C	718	49.0	934	62.8	<0.001
Number of altered cardiometabolic risk factors					<0.001
1	159	10.8	68	4.6	
2	427	29.1	357	24.0	
3	595	40.6	569	38.2	
4	285	19.4	494	33.2	

*chi-square test.

BP, blood pressure; HDL-C, high-density lipoprotein cholesterol.

**Table 3:** Prevalence of diabetic chronic complications.

Complications	Overall		Men		Women		P*
	N	n (%)	N	n (%)	N	n (%)	
DR	2850	596 (20.9)	1425	256 (18.0)	1425	340 (23.9)	<0.001
DN	2857	632 (22.1)	1429	313 (21.9)	1428	319 (22.3)	0.787
DPN	2856	902 (31.6)	1426	387 (27.1)	1430	515 (36.0)	<0.001
CVD	2836	356 (12.6)	1418	159 (11.2)	1418	197 (13.9)	0.036
Stroke	2834	260 (9.2)	1418	132 (9.3)	1418	128 (9.0)	0.795

*chi-square test.

DR, diabetic retinopathy; DN, diabetic nephropathy; DPN, diabetic peripheral neuropathy; CVD, cardiovascular disease.

Multiple logistic regression analysis revealed the relationships between cardiometabolic risk factors and the presence of diabetic chronic complications (Table 4). Among all the risk factors, elevated BP was significantly associated with all the chronic complications of diabetes except DPN (*P*<0.05). After adjusting for age, gender, duration of diabetes, and HbA1c, elevated BP was significantly associated with DR (OR 1.63; 95% CI: 1.22–2.19), DN (OR 3.16; 95% CI: 2.25–4.46), CVD (OR 2.71; 95% CI: 1.70–4.32) and stroke (OR 1.90; 95% CI: 1.15–3.12). Abdominal adiposity was significantly associated with DN (OR 1.39; 95% CI: 1.11–1.74). Elevated triacylglycerols was significantly associated with DR (OR 1.29; 95% CI: 1.05–1.58), and DN (OR 1.30; 95% CI: 1.05–1.58). Reduced HDL-C was significantly associated with stroke (OR 1.41; 95% CI: 1.05–1.88).

**Table 4:** Multiple logistic model for cardiometabolic risk factors for diabetic chronic complications.

	DR	DN	DPN	CVD	Stroke
Age	1.01 (0.99–1.02)	1.01 (1.00–1.02)*	1.02 (1.01–1.03)*	1.05 (1.04–1.06)*	1.07 (1.05–1.08)*
Duration of diabetes	1.09 (1.07–1.10)*	1.08 (1.06–1.09)*	1.06 (1.05–1.08)*	1.04 (1.02–1.06)*	1.02 (0.99–1.04)
Female gender	1.15 (0.93–1.41)	0.78 (0.64–0.96)*	1.24 (1.03–1.48)*	0.96 (0.74–1.23)	0.71 (0.54–0.95)*
HbA1c	1.04 (1.01–1.08)*	1.05 (1.01–1.08)*	1.03 (1.01–1.07)*	1.01 (0.94–1.06)	1.02 (0.92–1.03)
Abdominal obesity	1.19 (0.94–1.50)	1.39 (1.11–1.74)*	1.22 (0.99–1.50)	1.03 (0.77–1.37)	1.30 (0.95–1.79)
Elevated BP	1.63 (1.22–2.19)*	3.16 (2.25–4.46)*	1.18 (0.93–1.48)	2.71 (1.70–4.32)*	1.90 (1.15–3.12)*
Elevated triacylglycerols	1.29 (1.05–1.58)*	1.30 (1.05–1.58)*	1.04 (0.87–1.24)	1.08 (0.84–1.38)	1.04 (0.72–1.27)
Reduced HDL-C	1.01 (0.75–1.12)	1.19 (0.97–1.46)	1.02 (0.78–1.10)	1.25 (0.97–1.60)	1.41 (1.05–1.88)*
Elevated LDL-C				1.13 (0.94–1.20)	1.29 (0.95–1.74)

*P<0.05.

HbA1c, haemoglobin A1c; BP, blood pressure; LDL-C, low-density lipoprotein cholesterol; HDL-C, high-density lipoprotein cholesterol; DR, diabetic retinopathy; DN, diabetic nephropathy; DPN, diabetic peripheral neuropathy; CVD, cardiovascular disease.

Data are expressed as odds ratios with 95% confidence intervals.

Age, duration of diabetes, and HbA1c levels were modelled as continuous variables. Gender was a binary variable. Abdominal obesity, elevated BP, elevated triacylglycerols, reduced HDL-C, elevated LDL-C were binary variables that were defined according to the ATP III criteria.

## Discussion

We observed that the prevalence of altered cardiometabolic risk profiles was high among overweight or obese type 2 diabetes patients. Moreover, altered cardiometabolic risk factors, especially elevated blood pressure (defined as systolic/diastolic blood pressure ≥130/85 mmHg), were associated with several chronic diabetic microvascular and macrovascular complications in our study population.

Although the association of altered cardiometabolic risk factors with chronic complications of diabetes has not yet been investigated specifically in overweight and obese type 2 diabetic patients, our results were consistent with previous findings in type 2 diabetes patients in general. Elevated BP has been long recognized as a risk factor for DR in type 2 diabetes patients [15], [16]. The UK Prospective Diabetes Study (UKPDS) found that each 10 mm Hg decrease in systolic pressure was associated with significant reductions in risk of any type 2 diabetes-related complication (12%), death (15%), myocardial infarction (11%), and microvascular complications (13%) [17]. Moreover, the UKPDS reported that tight BP control (144/82 mm Hg), compared with less tight control (154/87 mm Hg), yielded significant reductions of 24% in risk of any clinical end point related to diabetes, 32% in deaths related to diabetes, 44% in strokes, and 37% in microvascular end points, predominantly owing to a reduced risk of retinal photocoagulation [18]. Interestingly, several recent studies reported an increased risk of CVD and stroke among type 2 diabetic patients that was associated with both high BP and too aggressive BP control [19], [20], [21]. Consistent with our findings, Isomaa et al. reported that a high HbA1c level was related to DPN, DR and DN [22]. Recently, a large multinational observational study of 66 726 patients with type 2 diabetes reported that obesity, systolic BP, and LDL-C were positively associated, while HDL-C was negatively associated, with diabetic macrovascular and microvascular complications [7]. However, in that study HbA1c was negatively associated with macrovascular complications, and the authors argued that this might have been due to the cross-sectional study design [7].

The prevalences of stroke, DR, DN and DPN observed in our study were higher than those reported in a previous study in type 2 diabetes patients who were not overweight (9.2% vs. 6.8%, 20.9% vs. 14.8%, 22.1% vs. 10.7% and 31.6% vs. 17.8%, respectively) [23]. The prevalence of CVD in our study (12.6%) was lower than that in the previous study (30.1%), probably because we did not include hypertension (prevalence of 64.5%) in our definition of CVD while they did. The higher prevalence of diabetic chronic complications may have resulted from a higher mean BMI in our population. It has been reported that each 5 kg/m^2^ increase in BMI was associated with about 30% higher overall mortality, 40% increase in vascular mortality [24] and a 5% increase in total stroke [25]. In addition, overweight at age 20 was associated with a significant threefold excess risk of chronic renal failure [26]. Therefore, weight control, in addition to glycemic control and BP control, should be taken into account. Additional studies are warranted to further clarify the relative importance of these risk factors in the prevention of chronic complications of diabetes.

This study has several limitations. First, it cannot provide evidence of causation between cardiometabolic risk factors and chronic complications of diabetes because it was a cross-sectional survey. Second, there were no normal-weight control patients. However, that did not affect the results obtained from the overweight or obese patients. Third, we included only patients who visited hospital clinics, which may have caused selection bias, because diabetes patients who are older, women, or have more complications tend to visit hospitals more often than others.

In conclusion, this multicentre, cross-sectional survey conducted in south China found a high prevalence of altered cardiometabolic risk profiles in overweight and obese type 2 diabetes. Altered cardiometabolic risk profiles, in particular elevated BP, were significantly associated with chronic complications of diabetes among the study patients. Future studies on the prevention of chronic complications of diabetes might focus on the effects of targeting blood pressure lowering. Randomized controlled trials are warranted to confirm the effects of blood pressure lowering therapy on the development of diabetic chronic complications.
